# Genomic analysis reveals the genetic diversity, population structure, evolutionary history and relationships of Chinese pepper

**DOI:** 10.1038/s41438-020-00376-z

**Published:** 2020-10-01

**Authors:** Shijing Feng, Zhenshan Liu, Yang Hu, Jieyun Tian, Tuxi Yang, Anzhi Wei

**Affiliations:** 1 College of Forestry, Northwest A&F University, Yangling, 712100 Shaanxi China; 2 College of Life Science, Northwest A&F University, Yangling, 712100 Shaanxi China; 3 Research Centre for Engineering and Technology of Zanthoxylum State Forestry Administration, Yangling, 712100 Shaanxi China

**Keywords:** Plant evolution, Population genetics

## Abstract

Chinese pepper, mainly including *Zanthoxylum bungeanum* and *Zanthoxylum armatum*, is an economically important crop popular in Asian countries due to its unique taste characteristics and potential medical uses. Numerous cultivars of Chinese pepper have been developed in China through long-term domestication. To better understand the population structure, demographic history, and speciation of Chinese pepper, we performed a comprehensive analysis at a genome-wide level by analyzing 38,395 genomic SNPs that were identified in 112 cultivated and wild accessions using a high-throughput genome-wide genotyping-by-sequencing (GBS) approach. Our analysis provides genetic evidence of multiple splitting events occurring between and within species, resulting in at least four clades in *Z. bungeanum* and two clades in *Z. armatum*. Despite no evidence of recent admixture between species, we detected substantial gene flow within species. Estimates of demographic dynamics and species distribution modeling suggest that climatic oscillations during the Pleistocene (including the Penultimate Glaciation and the Last Glacial Maximum) and recent domestication events together shaped the demography and evolution of Chinese pepper. Our analyses also suggest that southeastern Gansu province is the most likely origin of *Z. bungeanum* in China. These findings provide comprehensive insights into genetic diversity, population structure, demography, and adaptation in *Zanthoxylum*.

## Introduction

*Zanthoxylum* is an economically important aromatic genus of the Rutaceae family, which also includes *Citrus*^1–3^. This genus consists of ~250 species primarily native to warm temperate and subtropical regions across the world^4^. The fruits of *Zanthoxylum* have long been used in eastern Asian countries as a popular spice to improve taste, mainly due to the unique tingling oral sensation created by chemical compounds known as alkamides^5^. In addition to culinary applications, many species of *Zanthoxylum* have been used as traditional medicines with the functions of promoting blood circulation, regulating the meridians, removing cold dampness, strengthening teeth, and improving eyesight, as recorded in many classic monographs of medicine, including *ShenNongBenCaoJing* (Divine Farmer’s Classic of Materia Medica, the earliest traditional Chinese Medicine monograph written between ~200 and 250 C.E.) (Fig. S1), *Mingyi Bielu*, *Zhenglei Bencao*, and *Bencao Gangmu* (Compendium of Materia Medica)^6,7^. To date, *Zanthoxylum* fruits have been officially applied in more than 30 prescriptions for the treatment of numerous diseases^8–10^. Consequently, this genus has great potential for the development of industrial products and new drugs.

China is considered one of the modern diversity centers for the *Zanthoxylum* genus. Currently, China harbors at least 45 species and 13 varieties within this genus, of which *Zanthoxylum bungeanum* and *Zanthoxylum armatum* are the most popular species due to their wide range of cultivation and versatile applications (collectively referred to as Chinese pepper henceforth)^6,11^. *Z. bungeanum* is believed to be native to China and has a long history of cultivation beginning in the Jin Dynasty (265–420 C.E.)^6,12^. *Z. armatum* is also known as Indian prickly ash or toothache tree and is mainly distributed in Southwest China^13^. Chinese pepper was first described nearly 2600 years ago in *Shijing* (Classic of Poetry), which recorded that in the period of the pre-Qin Dynasty (before 221 B.C.), the fruits of Chinese pepper were regarded as precious tokens and used to worship ancestors and pray for more children or a good harvest^12^. The earliest cultivation of Chinese pepper probably occurred in Sichuan at least 1500 years ago, where an abundance of improved varietal resources were available (Supplementary Fig. 1). It can also be concluded from the scarce historical written record and excavated fruits that, before cultivation, the main distribution of wild ancestral populations of Chinese pepper covered the mountain regions of Sichuan, Gansu, Shaanxi, Henan, Hubei, and Hunan provinces. However, from the Tang Dynasty to the Ming Dynasty, the cultivation regions of Chinese pepper extended nationwide, and at present, the wild populations have largely retreated into marginal regions or been replaced by cultivated populations^6,12^.

Although numerous landraces and elite cultivars potentially locally adapted to nationally diverse habitats have been developed via thousands of years of natural and artificial selection, domestication, and evolutionary processes (Supplementary Fig. 2), how these processes shaped the current pattern of genetic variation within and between populations remains obscure. A range of previous studies attempted to study the genetic diversity of specific local varieties or species with molecular markers such as isozyme^14^, random amplified polymorphic DNA^15,16^, inter simple sequence repeat^15,17^, amplified fragment length polymorphism^18^, simple sequence repeat^19–22^, and chloroplast DNA^23,24^ markers. The limited number of polymorphic loci produced by these low-resolution markers hinders genetic research on *Zanthoxylum* species. Therefore, a detailed investigation of the genetic structure, origin, and evolutionary history of these two cultivated Chinese pepper species is essential for the conservation and management of germplasm resources, breeding improvement, tracking crop varieties, and genomic selection. Herein, we present comprehensive analyses based on the genome-wide variation identified via the genotyping-by-sequencing (GBS) approach in a collection of 112 *Z*. *bungeanum* and *Z*. *armatum* accessions from 43 cultivars/populations from the current range to resolve key questions in *Zanthoxylum* evolution.

## Results

### Population structure and variation characteristics

**Fig. 1:**
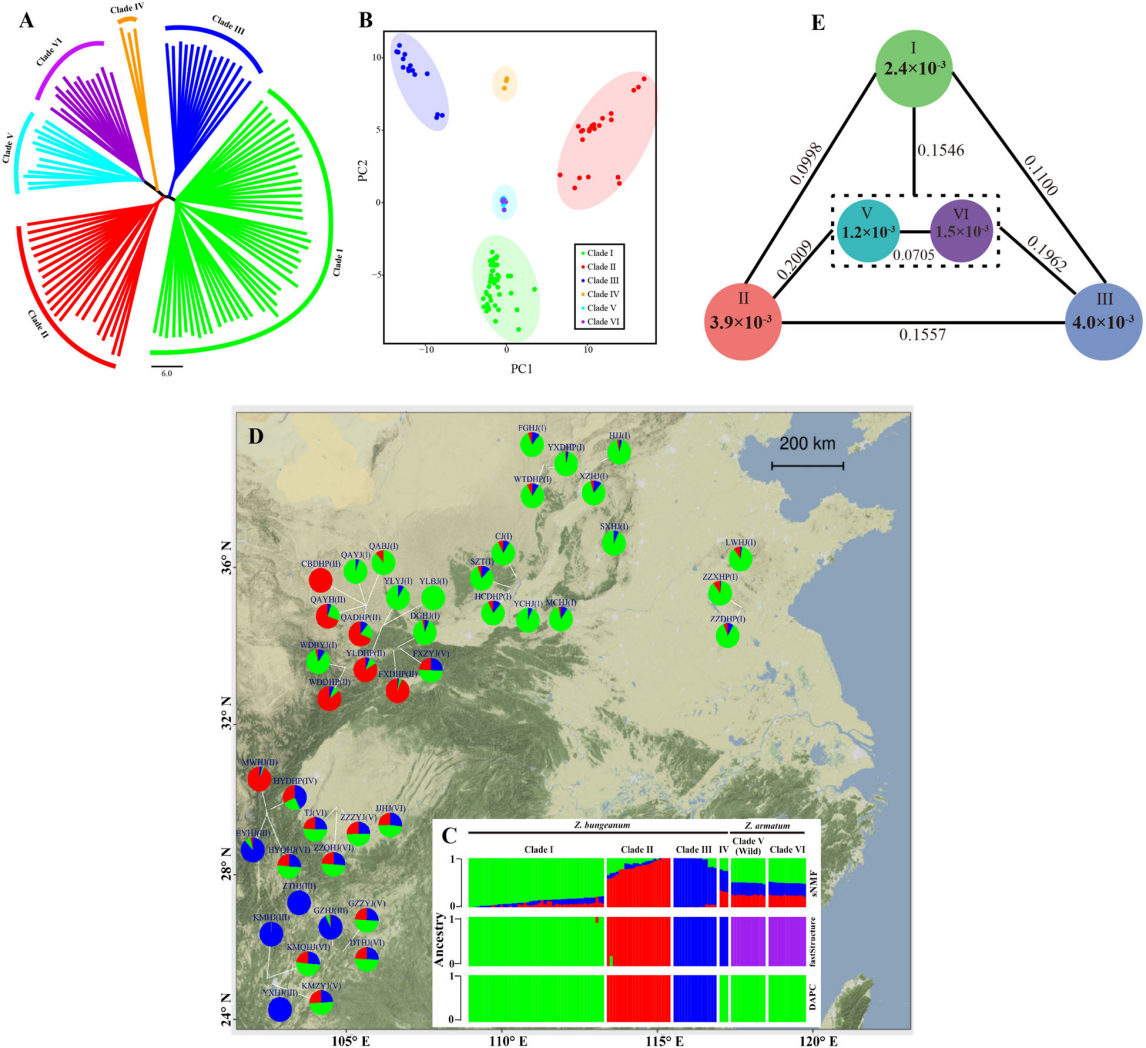
Genetic structure and geographic distribution of Chinese pepper in China. **a** Phylogenetic tree of 112 *Z. bungeanum* and *Z. armatum* accessions constructed from 38,395 SNPs. Branches of the tree are highlighted by different colors. For the *Z. bungeanum* accessions, colors correspond to the following groups: green, Clade I; red, Clade II; blue, Clade III; and orange, Clade IV. For the *Z. armatum* accessions, colors correspond to the following groups: cyan, wild; and purple, cultivated. **b** PCA plots of the first two principal components (PCs) for all 112 accessions. PC1 and PC2 differentiated the *Z. bungeanum* accessions from each other, whereas the *Z. armatum* accessions remained tightly clustered and shared similar genetic distances to each *Z. bungeanum* clade. **c** Population structure revealed by the sNMF, fastSTRUCTURE, and DAPC methods. Each individual is represented by a vertical (100%) stacked column of genetic components, with proportions shown in color for the best *K* = 3 or 4. **d** Map showing the diversity in structure of genomic SNPs, based on the average *Q* value for each cultivar/population, from runs of sNMF at *K* = 3 (see Table S1 for cultivar/population codes). **e** Nucleotide diversity (*π*) and population divergence (*F*_*ST*_) across the five major clades. The values for each circle represent a measure of nucleotide diversity for each clade, and the values on each line indicate population divergence between two clades

The final dataset consisted of 38,395 biallelic single nucleotide polymorphisms (SNPs) genotyped for 112 accessions, including 87 *Z*. *bungeanum* accessions, 13 cultivated *Z*. *armatum* accessions, and 12 accessions of *Z*. *armatum* from natural populations (Fig. 1d and Supplementary Table 1). Distance-based clustering by neighbor joining revealed a clear split between the *Z*. *armatum* and *Z*. *bungeanum* accessions. The clustering results also provided strong support for the subdivision of 25 *Z*. *armatum* accessions into two distinct genetic clades corresponding to wild and cultivated lineages (Clades V and VI hereafter) and 87 *Z*. *bungeanum* accessions into four clades (Clades I, II, III, and IV hereafter; Fig. 1a), largely reflecting their geographic distributions. Principal component analysis (PCA) performed with all loci resulted in four major clades for the *Z*. *bungeanum* accessions, as suggested by the neighbor-joining tree; however, the *Z*. *armatum* accessions remained tightly clustered and shared a similar genetic distance to each *Z*. *bungeanum* clade (Fig. 1b), following a trajectory suggestive of admixture between species. Genotypic clustering using sparse nonnegative matrix factorization (sNMF), fastSTRUCTURE, and discriminant analysis of principal components (DAPC) suggested that *a K* value of 3 or 4 represented a feasible model of the data. All approaches identified the same first three genetic clades as did PCA, but they differed in their ability to resolve the relationship between the last three clades and the first three clades. Accessions of Clades IV, V, and VI were identified as admixture hybrids of Clades I, II, and III by sNMF. However, we found no sign of hybridization between the 2 species using fastSTRUCTURE, since none of the 112 sampled accessions showed intermediate genotypes. Using the DAPC nonmodel-based assignment method, all individuals of Clades IV, V, and VI were assigned to Clade I (Fig. 1c).

Nucleotide diversity (*π*) analysis showed that Clades II and III of *Z. bungeanum* had the highest genetic diversity, followed by Clade I. The genetic diversity of Clades II and III was threefold higher than that of *Z. armatum* clades (Fig. 1e). We also calculated the genetic differentiation statistics (*F*_*ST*_) between the five major *Zanthoxylum* clades and found substantially higher genetic differentiation between *Z*. *bungeanum* clades (*F*_*ST*_ = 0.0998−0.1557) than between *Z*. *armatum* clades (*F*_*ST*_ = 0.0705; Fig. 1e). The lowest *F*_*ST*_ supported a closer relationship between *Z. armatum* and Clade I rather than Clade II or III. In addition, we found that all *Z*. *bungeanum* accessions possessed higher levels of heterozygosity than the *Z*. *armatum* accessions (Supplementary Fig. 3), despite the two species sharing much higher levels of heterozygosity than their close relative *Citrus* in the Rutaceae family^1^.

### Demographic history and correlation with climate change

**Fig. 2:**
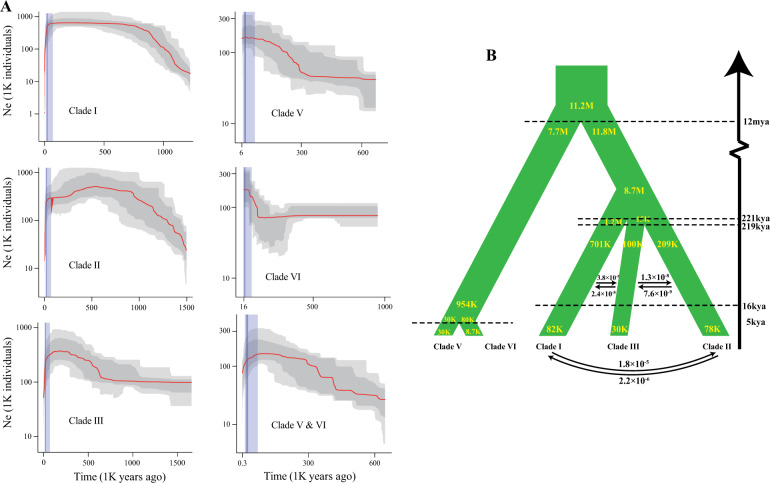
Demographic history of *Z. bungeanum* and *Z. armatum*. **a** Historical effective population size (*N*_*e*_) for *Z. bungeanum* (left) and *Z. armatum* (right) clades from ~1.8 million years ago (mya) to present. The *N*_*e*_ of each clade was rescaled using the generation time *g* = 4 years and mutation rate per year *μ* = 2.6 × 10^−9^; ref. ^20^. The stairway plot shows that *Z. bungeanum* clades have undergone an apparent steep decline in *N*_*e*_ since the LGM, whereas the LGM did not result in population declines in *Z. armatum* clades. Light blue represents the last glacial period (70−11.5 thousand years ago, kya), and the dark blue shadow represents the LGM (26−18 kya). Data are median estimates from 200 bootstrap replicates (black line), 95% confidence intervals (light shading), and 75% confidence intervals (dark shading). **b** Schematic model of a demographic scenario based on population splitting and changes in *N*_*e*_ revealed by fastSIMCOAL2

Generally, overall skews in the site frequency spectrum (SFS) reflect signatures of past demographic events. Here, we used folded SFS derived from each clade and the model-flexible stairway plot method^25^ to infer fluctuations in the effective population size (*N*_*e*_) over time for each clade separately. The stairway plot revealed that clades from the same species shared highly similar patterns of historical fluctuation in *N*_*e*_ over time that roughly mirrored known events of environmental upheaval. Each of the *Z*. *bungeanum* clades underwent long-term steady population expansion during the early-to-mid-Pleistocene era (~1.8–0.13 million years ago (mya)), although the patterns differed slightly, followed by an apparent steep decline in *N*_*e*_ after the Last Glacial Maximum (LGM) (Fig. 2a, left). Clades V and VI of *Z*. *armatum* maintained relatively fast population growth beginning 300 and 120 thousand years ago (kya), respectively, until the LGM slowed down or prevented that process (Fig. 2a, right). Reconstruction of the more recent demographic history of *Z*. *armatum* clades was impossible using the stairway plot approach due to the very limited amount of variation in this species. Nonetheless, analysis of a combination of Clades V and VI revealed a twofold decline in *Z*. *armatum N*_*e*_ after the LGM (Fig. 2a, right, Clades V and VI). These results suggested that the dramatic climate shifts during the Pleistocene may have been a considerable demographic driver of the recent growth and decline of *Zanthoxylum* populations.

Results from species distribution modeling (SDM) (Supplementary Fig. 4) may provide more direct evidence for the abovementioned disparity. We observed high environmental (climatic) niche overlap for these two species, meaning it is unlikely that they underwent adaptive divergence. This was also supported by the outlier locus analysis, which detected only one locus as being potentially under divergent selection between species (see below). However, the SDM supported the hypothesis of a dynamic historical distribution for *Zanthoxylum*, since the estimated potential distributions of both species have changed over the past 130 kya. For *Z*. *bungeanum*, we observed a notable extensive collapse in suitable habitats from the LGM to mid-Holocene and somewhat of a recovery at present. For *Z*. *armatum*, we found a slight contraction in a suitable distribution area during the LGM and extensive habitat recovery from the mid-Holocene to the present day. The dynamic ranges of these two species supported the idea that the formation of these genetically distinct lineages may be due to periodic fragmentation, possibly isolation events derived from climate change.

Using the joint SFS and Markovian coalescent approximation approach implemented in fastSIMCOAL2^26^, we simulated more recent demographic fluctuations in population size and inferred the time of the divergence events between species and between clades. The results of fastSIMCOAL2 analysis for *Z*. *armatum* (Fig. 2b, Supplementary Fig. 5) were consistent in the sense that only three models (Models 9, 11, and 12; Supplementary Fig. 5), all characterized by isolation with exponential shrinkage in cultivated *Z*. *armatum*, exhibited any appreciable model probability. The demographic model in which *Z*. *armatum* diverged through a dynamic process involving exponential decline in population size in cultivated *Z*. *armatum* but constant population size in wild *Z*. *armatum* (Model 9) produced a much better fit than alternative models. The isolation-with-migration model with asymmetric migrations and exponential population decline in cultivated *Z*. *armatum* (Model 11) and the model assuming exponential decay in both wild and cultivated *Z*. *armatum* without extensive postdomestication gene flow (Model 12) generated similar log-likelihoods given the data but lower Akaike information criterion (AIC) scores due to having additional parameters. Assuming a mutation rate of 2.6 × 10^−9^ substitutions per site per year^20^ and 4 years per generation, we estimated a split time of ~5440 years ago and a 200-fold decline in *N*_*e*_ for cultivated *Z*. *armatum* (Fig. 2b).

The fastSIMCOAL2 analysis of divergence among the three genetic clades of *Z. bungeanum* revealed that the model in which the split between Clades II and III occurred shortly after they diverged from Clade I (Model 13) was the optimal model, with a maximum log-likelihood value of −21,074. This model supported the results of stairway plot analysis in terms of the demographic decline during the LGM for all three clades (Fig. 2b, Supplementary Fig. 5). This simulation showed that Clades I and II/III diverged 221 kya with a 95% confidence interval (CI) of 212−229 kya, immediately followed by divergence between Clades II and III 219 kya (95% CI = 210–226 kya), indicating that the split events occurred almost simultaneously. Simulation of divergence between *Z*. *armatum* and *Z*. *bungeanum* showed that the model without migration after population splitting had the highest model probability. This model predicted that *Z*. *armatum* and *Z*. *bungeanum* diverged ~12 mya (95% CI = 10–14.4 mya; Fig. 2b).

### Genomic relationships between cultivars

**Fig. 3:**
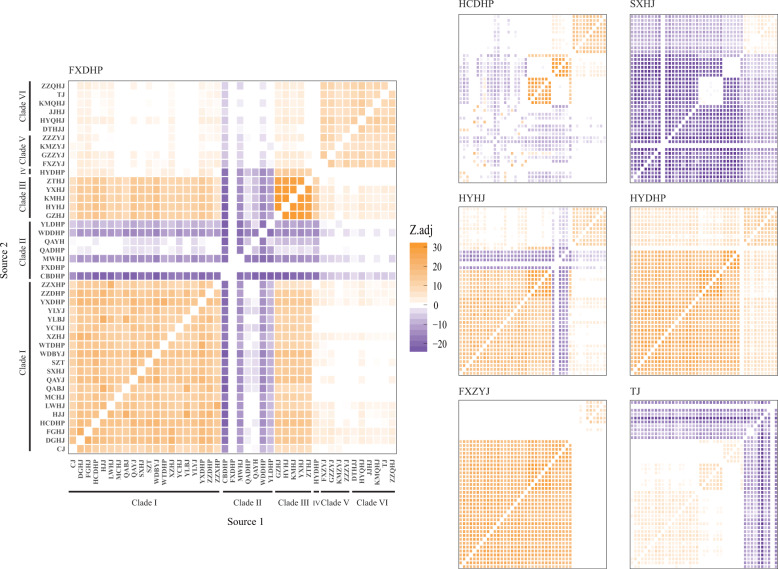
Heat maps for three-population *f*3 test statistics. Introgression is significant if the false discovery rate-adjusted *Z*-score is significantly negative (adjusted *Z*-score ≤ 1.96, purple)

Hybridization between *Zanthoxylum* cultivars is relatively unexplored. Here, explicit allele-sharing analysis by three-population admixture-*f*3 tests detected no traces of interspecific admixture; however, we detected a sizable amount of intra- and interclade admixture within species, which suggested ongoing and possibly bidirectional gene flow between clades and deep divergence between species (Fig. 3). Most Clade I cultivars appeared to be highly admixed, including QAYJ, SXHJ, SZT, CJ, WTDHP, YCHJ, YLBJ, YLYJ, MCHJ, and ZZDHP from northern China, indicating multiple waves of migration of *Zanthoxylum* genetic resources into this region. HYDHP, FXZYJ, and QAYH displayed no signatures of introgression from other cultivars, regardless of geographical proximity. ZZXHP, YXDHP, and Clade III cultivars received gene flow from one or more sources in relatively close proximity, indicating a pattern of genetic isolation by distance for these cultivars.

**Fig. 4:**
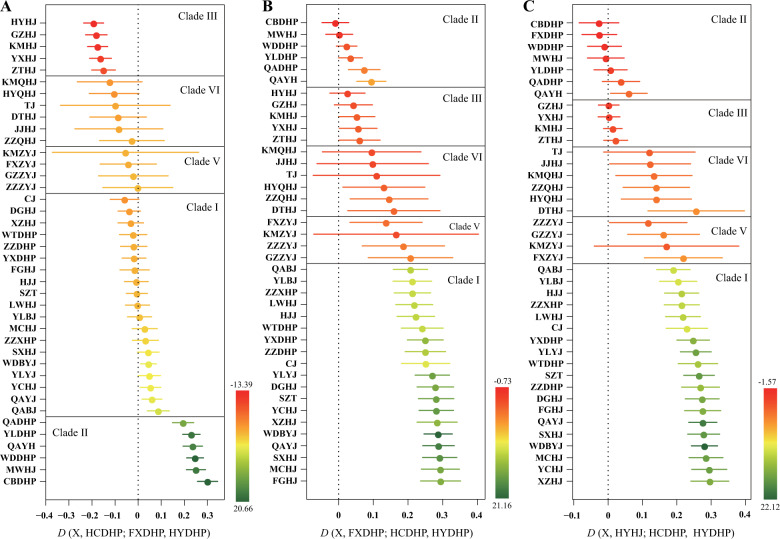
Genetic affinity between each of the three elite *Zanthoxylum* cultivars (HCDHP, FXDHP, and HYDHP) and another cultivar (X) measured by *D*-statistic testing of the forms. **a** *D*(X, HCDHP, FXDHP, HYDHP), **b** *D*(X, FXDHP, HCDHP, HYDHP), and **c** *D*(X, HYHJ, HCDHP, HYDHP) (points), using HYDHP as an outgroup. Error bars represent approximately three standard errors (*p* = ~0.001)

To assess the genome-wide affinities of the top-producing cultivars FXDHP, HCDHP, and HYHJ, we performed outgroup-*f*3 tests of the form *f*3 (HYDHP; FXDHP/HCDHP/HYHJ, X). We found that FXDHP was most closely related to CBDHP and MWHJ from Gansu and Sichuan, respectively. HCDHP shared more drift with QAYJ and WDBYJ in Gansu, MCHJ in Henan, and SXHJ in Hebei than any other native cultivars in Shaanxi. HYHJ showed higher levels of allele sharing with ZTHJ and YXHJ in Yunnan (Supplementary Fig. 6). In addition, we observed similar affinities using *D*-statistics (Fig. 4).

**Fig. 5:**
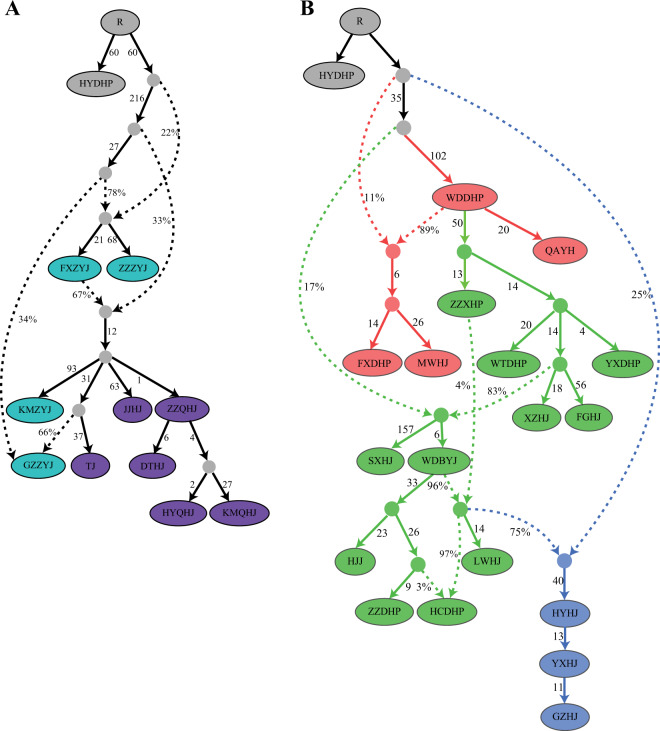
Admixture graph of the history of *Zanthoxylum* cultivars. Models of phylogenetic relationships among *Z. armatum* (**a**) and *Z. bungeanum* (**b**) cultivars augmented with admixture events. Drift for each lineage is given in units proportional to 1000 × *F*_*ST*_, and admixture events (dotted lines) are denoted by the percentage of ancestry. At least three and five admixture events are inferred in the history of *Z. armatum* and *Z. bungeanum* cultivars, respectively. Inferred ancestral populations are indicated by filled circles, and lineages descending from them are colored as follows: Clade I (green), Clade II (red), Clade III (blue), Clade V (cyan), and Clade VI (purple)

To construct an explicit model for the settlement of each *Zanthoxylum* cultivar, we used *qpGraph* from the ADMIXTOOLS package to integrate the observed signals of gene flow into a single historical admixture graph (AG) framework by fitting empirical and predicted *f*-statistics. AGs are generalizations of phylogenetic trees combined with admixture events, which are believed to be powerful tools for revealing a more complex history than can be captured by a simple tree-like topology because AGs predict all patterns of *f*-statistics (including *f*_2_, *f*_3_, and *f*_4_) that can be used to assess the fit of a proposed evolutionary scenario. Figure 5a presents an AG corresponding to all *Z*. *armatum* cultivars that was a good fit to the data, as none of the predicted *f*-statistics were more than three standard errors from the observed values (max|*Z*| = 2.776). Using this model, we observed a highly reticulated relationship among cultivars of Clade V, whereas all cultivars of Clade VI descended from a putative ancestral population derived from an admixture event between FXZYJ and a more ancient ancestor. Figure 5b presents the best-fitting model for the history of 19 selected *Z*. *bungeanum* cultivars, despite the largest |*Z*|-score for differences between observed and predicted *f*-statistics reaching 5.52 among the 14,706 statistics we tested. This indicated that more subtle gene flow events were needed to accommodate the data. However, this model provided a better statistical fit to the data than the tree shown in Fig. 1a and led to several novel inferences.

First, the above result was consistent with the divergence pattern of *Z*. *bungeanum* revealed by fastSIMCOAL2 and indicated that southeastern Gansu (i.e., the WDDHP group) was the most likely origin of this species. Second, FXDHP and MWHJ, located in the opposite direction of WDDHP, have experienced very little genetic drift since divergence from their common ancestral population shared with WDDHP and formed a branch, suggesting recent anthropogenic contributions to the migration of this clade in this region. Third, we observed multiple streams of origin-to-north gene flow that might have contributed substantially to the genetic diversity in Clade I. Most Shanxi populations (including WTDHP, YXDHP, XZHJ, and FGHJ) descended entirely from a single ancestral population shared with WDDHP and formed a simple cluster. The direct migration from the origin to Shandong without an intermediate zone was in accordance with the historical record that business activity promoted the spread of cultivation practices in this region no later than 1400 years ago (*Qimin Yaoshu*). We also inferred back-migration of populations related to WDBYJ from Shanxi to the origin. WDBYJ provided several strands of ancestry that spread to northern China, including the formation of LWHJ and ZZDHP (both in Shandong) as well as HCDHP (Shaanxi). Finally, we concluded that Clade III populations derived large proportions of their ancestry from both Clades I and II, namely, an estimated 25% from one basal population of Clade I and 75% from a population closely related to WDBYJ. This conclusion was consistent with the extensive gene flow from Clade I to Clade III revealed by fastSIMCOAL2.

### Selection scan

**Fig. 6:**
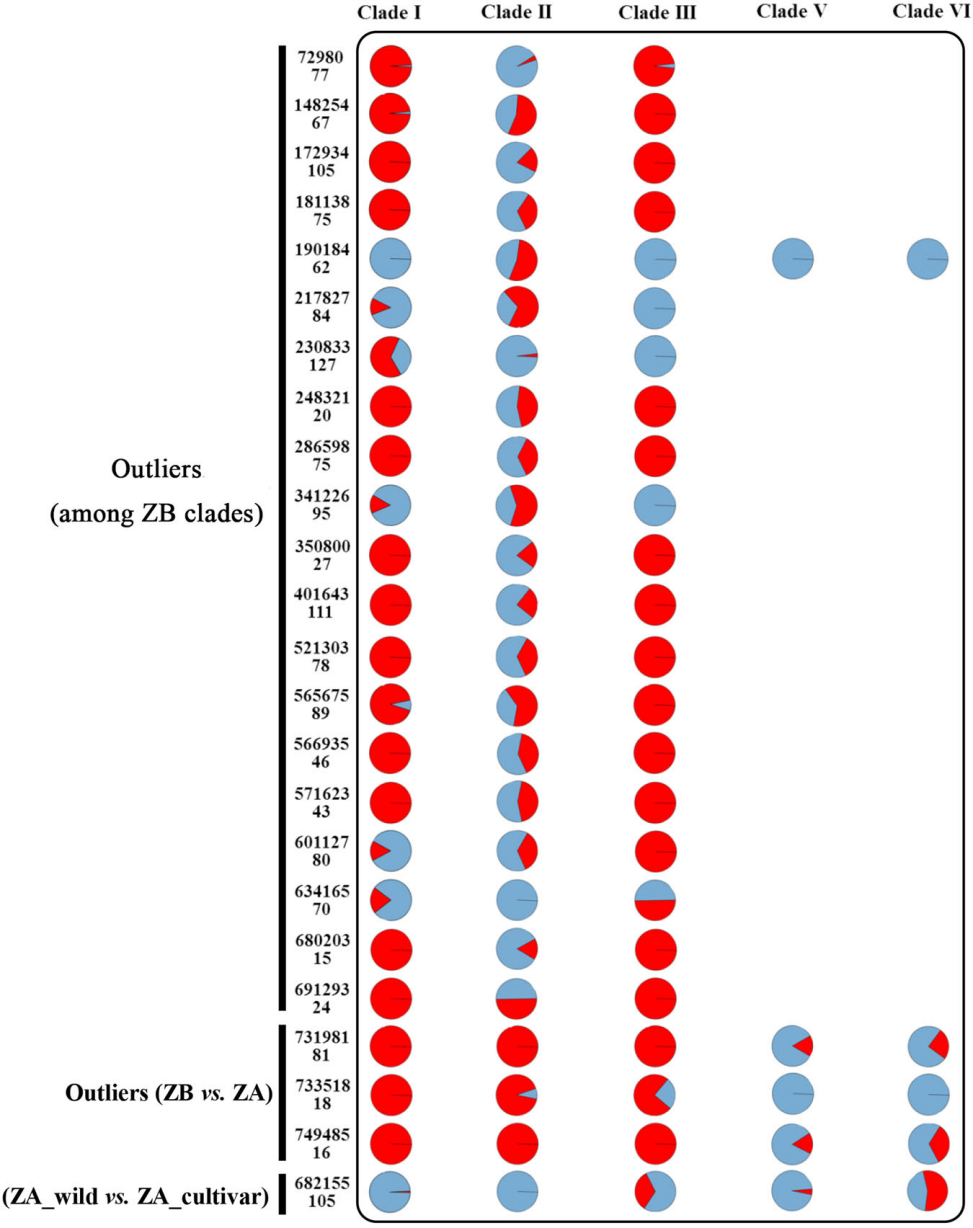
Frequencies of outlier loci in each clade of *Z. armatum* and *Z. bungeanum*. SNPs are identified by the locus number (top left) and SNP position within the locus in base pairs (bottom left). The first 20 loci are outliers between *Z. bungeanum* cultivars, the next three are outliers between *Z. bungeanum* and *Z. armatum*, and the last one is an outlier between wild and cultivated accessions of *Z. armatum*. ZA *Z. armatum*; ZB *Z. bungeanum*

Multiple outlier tests (Arlequin, FLK, LEA, and OutFLANK) identified a set of 24 outlier loci with higher *F*_*ST*_ values than expected (Fig. 6). Twenty of these loci showed evidence of spatially diversifying selection among clades within *Z*. *bungeanum*, whereas only three showed evidence of spatially diversifying selection between wild and cultivated clades within *Z*. *armatum*. There was only one locus displaying evidence of divergent selection between species. We obtained the flanking regions of all outliers, four of which matched protein-coding genes. We detected three nonsynonymous and four synonymous mutations at locus 682155 within the IQ-Domain 1 gene. This gene is involved in resistance against herbivory by generalist chewing and phloem-feeding insects^27^, one of the traits that differs between wild and cultivated cultivars. Locus 230833 has a nonsynonymous mutation and a nonsense mutation within the cytokinin riboside 5′-monophosphate phosphoribohydrolase LOG7 gene, which encodes a protein that plays a central role in cytokinin activation in plants. Cytokinin signaling is of pivotal importance in regulating various processes in plant growth and development, including vascular development^28,29^, wood formation^30^, normal primary root growth^25^, and female gamete and embryo development^31^. It also plays a role in regulating adaptive responses to abiotic stresses such as cold and drought^32,33^. These candidate genes may provide clues for further research on the adaptation of Chinese pepper cultivars to biotic and abiotic stress variations and are certainly worthy of more targeted analysis, despite the phenotypic correlates of clinal variation in some genes remaining unclear.

## Discussion

Chinese pepper, also known as “Huanjiao,” is an economically important tree plant widely used as a spice and medicine with a national distribution in China. Unraveling the genomic relationships and evolutionary history of Chinese pepper can help clarify the interspecific admixture and gene flow between various cultivars, as well as infer the origin of domestication and pattern of historical fluctuation in effective population size. To the best of our knowledge, this is the first report to comprehensively study the genetic relationships and demographic history of a collection of *Z. bungeanum* and *Z. armatum* accessions using next-generation sequencing.

Based on PCA, phylogenetic analysis and genetic structure analysis, we believe that *Z. bungeanum* and *Z. armatum* have clearly split without secondary contact, inhibiting the formation of reproductive isolation. Moreover, we found strong evidence for splitting into at least four genetic clades within *Z. bungeanum*, corresponding to the geographic distribution from these combined analyses, including genetically distinct clades that contain only individuals from the HYDHP cultivar. In contrast, *Z. armatum* accessions are separated into wild and cultivated groups. These results are consistent with our previous report that the Qinling Mountains were the main geographic barrier for gene flow between *Z. bungeanum* clades and that cultivated and wild *Z. armatum* accessions exhibited significant genetic differentiation^34,35^. How does such strong genetic structure occur in a domesticated species of plant? First, *Z. bungeanum* covered a wider distribution range across China than *Z. armatum*. Plant populations with a large distribution tend to form subpopulations due to environmental heterogeneity, genetic variation, and restricted gene flow, which are all found in *Z*. *bungeanum* and may have led to its strong genetic differentiation. Second, as local adaptation plays an essential role in maintaining production in traditional agricultural systems, selection for adaptation to each region’s environment and monocultures of genetically similar individuals formed regionally by farmers’ seed selection increases overall genetic diversity but leads to little to no genetic variation within local cultivars. Thus, the strong differentiation between *Z. bungeanum* clades likely reflects the effects of both geographical isolation due to the Qinling Mountains as well as a long period of divergent domestication.

We also showed that the nucleotide diversity of *Z. bungeanum* was threefold higher than that of *Z. armatum*, which was probably attributed to the restricted distribution and thus limited sampling of *Z. armatum* and otherwise the frequent local or long-distance exchange of seeds of *Z. bungeanum* by farmers during long-term traditional agricultural practices. Puzzlingly, although the domesticated accessions of *Z. armatum* were clearly genetically differentiated from wild ones, they still possessed variation even higher than that of wild varieties. This result suggests that the population size bottleneck at domestication only slightly reduced genetic variability (i.e., a large number of individuals must have been selected for initial domestication, or domestication must have occurred simultaneously in multiple locations). Consistent with our previous study of the transcriptome^20^, Chinese pepper presents a higher level of heterozygosity (Supplementary Fig. 3) than other highly heterozygous plants, such as *Citrus*^1,3^ and pistachio^36^, indicating that Chinese pepper may have undergone extensive inter- or intraspecific hybridization. In summary, such patterns probably reflect a complex history of population subdivision in Chinese pepper.

To explore the demographic histories of *Z*. *bungeanum* and *Z*. *armatum*, we inferred effective population size changes, dynamic distributions, and divergence events between species and between clades. We detected continuous population expansion of ~1.8–0.13 mya in the *Z. bungeanum* clades, followed by an apparent population decrease after the LGM. However, we observed that the *Z. armatum* clades showed a very different pattern from those of *Z. bungeanum*, with continuing increases in *N*_*e*_ from 300 or 200 kya until the LGM. The results of environmental (climatic) niche modeling paralleled the population size change patterns of both species. It is noteworthy that the relatively cold and dry climate of Marine Isotope Stage 4 (58–74 kya, as indicated by the mass accumulation rate of Chinese loess^37^) and the LGM did not result in population declines in either of the *Z*. *armatum* clades but did have a strong negative effect on *Z*. *bungeanum*. One potential explanation for this apparent disparity is that the *Z*. *armatum* populations we analyzed were mainly from mountain regions in Southwest China, one of the world’s major plant diversity hotspots^38^. This region is thought to have harbored refugia during the LGM because organisms survived there and recolonized (radiated) outwards during interglacial periods^39^. The proposal that the topographical complexity of this region may be a crucial buffer against climate change was further confirmed by the unequal decay in *N*_*e*_ of *Z*. *bungeanum* clades during the LGM. The LGM resulted in a ~660- and 100-fold collapse in Clades I and II, respectively, both of which were mainly distributed in northern or northwestern China. In contrast, we observed only a sixfold decrease in *N*_*e*_ for Clade III during the LGM, which was largely sympatric with *Z*. *armatum*. This result indicates that plants sharing the same habitat might have similar responses to climate change and thus similar demographic histories.

The protracted increase in *N*_*e*_ for *Zanthoxylum* clades before the LGM raises the question of its causes. One possibility is that the expansion of *N*_*e*_ might be attributed to the high heterozygosity of these Chinese pepper clades (Supplementary Fig. 3). The heterozygosity in *Z. bungeanum* was higher than that in *Z. armatum*, which is in accordance with the wider distribution and larger contemporary *N*_*e*_ of the former. Note that both Chinese pepper species exhibited higher heterozygosity and a larger *N*_*e*_ compared with those of their close relative mandarin^40^. A second possibility is that the expansion reflects a natural process that operates in Chinese pepper progenitor populations. For example, recurrent episodes of harsh climatic conditions have forced the ancestral populations of Chinese pepper into ongoing cycles of retreating into and re-expansion from numerous isolated refugia, thus expediting diversification. By the time of the LGM, a period of extremely low temperature, both *Z. armatum* and *Z. bungeanum* had experienced precipitous declines, which was also the case in many plant species that have experienced fluctuations during Pleistocene climatic shifts. Both long-lived perennials, such as silver birch^41^, poplar^42^, grape^43^ and orange^40^, and annual plants, including Arabidopsis^44^, experienced a period of dramatic decline in *N*_*e*_ in this period. If the LGM caused a population decrease in Chinese pepper, one might expect to observe population recovery of Chinese pepper after glacial periods. Surprisingly, we detected no evidence for post-LGM expansion of Chinese pepper. This observation contrasts sharply with findings for peach^45^, maize^46^, and African rice^47^, all of which had largely recovered following the LGM. We hypothesize that the lack of expansion in Chinese pepper is attributable to the dynamics of perennial demographic trends, specifically the short time frame (in generations) and clonal propagation. The feature of demographic inference from grape is consistent with our hypothesis, but wild grape also had a greater than fivefold *N*_*e*_ increase following the LGM.

In our study, we speculate that the divergence time of *Z*. *armatum* and *Z*. *bungeanum* was ~12 mya (Fig. 2b). This inferred timescale is consistent with the estimate based on two plastids and two nuclear markers^27^ and corresponds to the Middle Miocene disruption, a steady period of cooling that expanded ice sheet volumes globally^48^. The results from the analysis of historical divergence between *Z*. *armatum* clades also provided important clues regarding the domestication history of Chinese pepper, although wild accessions of *Z*. *bungeanum* were not available in the current study. The split between wild and cultivated clades occurred ~5 kya, long before the first record of Chinese pepper in *Shijing* (Classic of Poetry), which was written 2600 years ago. The demographic analysis of *Z*. *bungeanum* showed that emergence and diversification occurred ~220 kya, long before human agricultural activity. These results suggested multiple origin centers for the domestication of Chinese pepper and weak selection pressure during this process. The fastSIMCOAL2 analysis also revealed that the timescale of divergence between Clades I and II/III coincided with the onset of the Penultimate Glaciation (0.3–0.13 mya), one of the most extensive Pleistocene glaciations in China that resulted in large ice caps and extremely large valley glaciers^37^. More specifically, this method detected severe bottlenecks for each clade during this period that were not observed in the stairway plot analysis. These findings are largely in line with expectations if climate change has been a major driver of variation in *Z*. *bungeanum* population size. The association of rapid divergence with key glacial periods suggests, but does not confirm, that *Z*. *bungeanum* divergence was driven by glacial cycles that resulted in direct habitat fragmentation, with adjacent refugia separated by glacial fingers.

Chinese pepper is among the earliest domesticated crops, with several thousands of years of cultivation history. Numerous varieties of Chinese pepper have been cultivated during the long-term domestication process^6^. Some were developed locally, and others were introduced. Distinct regional demands for different cultivars reflect local idiosyncrasies in consumer tastes; for instance, the cultivars of *Z. bungeanum* distributed within the tropical and subtropical regions south of the Qinling Mountains contain more numbing components but fewer leaf glandular puncta than those north of the Qinling Mountains^35^. We assessed the affinities between each variety/cultivar pair using three approaches. Based on outgroup-*f*3 tests and *D*-statistics, cultivars distributed in the vicinity of the Qinling Mountains showed the closest genetic relationships, while those far from the Qinling Mountains displayed introgression from one or more cultivars in relatively close proximity. These results indicated that some elite cultivars have been introduced and cultivated in many other areas across China. Nonetheless, there is no evidence of recent admixture between species because of probable reproductive isolation. In addition, we speculate that Gansu is the most likely origin of *Z*. *bungeanum*, which is in accordance with the earliest historical record of Chinese pepper in *Qimin Yaoshu*^12^.

## Conclusions

This is the most comprehensive survey of the evolutionary history of Chinese pepper to date. We identified at least three genetic clades within *Z. bungeanum* corresponding to their geographic distributions across China. Domesticated *Z. armatum* accessions (Clade VI) were found to be genetically differentiated from wild ones (Clade V), but they maintained high levels of genetic variability, suggesting a short domestication event involving a large number of individuals. This may also hold true in *Z. bungeanum* because we observed a relatively large effective population size in each clade of *Z. bungeanum*.

Based on our results, we can outline a model for the early evolutionary history of Chinese pepper. We infer that WDDHP (Gansu) was the ancestral population of *Z. bungeanum*, and the divergence among *Z. bungeanum* clades occurred ~200 kya. Wild accessions of *Z. armatum* clustered together and possibly diverged from cultivated accessions ~3−5 kya. Moreover, our results suggest that climate change drove an apparent steep decline in the effective population size of *Z. bungeanum* clades, rather than *Z. armatum* clades, during the LGM. Further insights into the history of *Zanthoxylum* will benefit from analyses similar to those performed here on whole genome sequences and from the collection of data from more populations of *Zanthoxylum* taxa. This could greatly aid in the curation of the many misidentified accessions and accelerate molecular breeding efforts for cultivated *Zanthoxylum* species.

## Materials and methods

### Material sampling and DNA extraction

Based on field investigations and with the permission of private landowners, we collected 87 *Z*. *bungeanum* accessions and 13 *Z*. *armatum* accessions from 39 cultivars in their native range throughout China. In addition, 12 wild accessions of *Z*. *armatum* from four natural populations were sampled in Yunnan, Guizhou, Sichuan, and Shaanxi provinces (Fig. 1d, Table S1). Young, healthy and fresh leaves were stored in silica gel for genomic DNA extraction. Total DNA was isolated using a DNeasy Plant Mini Kit (Tiangen Biotech., Beijing, China). Subsequently, the DNA degradation and contamination of each sample were monitored on 1% (w/v) agarose gels, and DNA purity was checked using a NanoPhotometer^®^ spectrophotometer (IMPLEN, CA, USA).

### Genotyping-by-sequencing (GBS) library preparation and sequencing

The protocol used to obtain the GBS library involved three major steps: restriction digestion, ligation, and PCR amplification. DNA concentration was measured using a Qubit^®^ DNA Assay Kit in a Qubit^®^ 2.0 Fluorometer (Life Technologies, CA, USA). First, a predesign experiment was performed to select the best restriction enzyme and sizes of restriction fragments. To maintain the sequence depth uniformity of different fragments, a narrow length range was selected (~50 bp). Afterwards, the GBS library was constructed according to the predesigned scheme. Genomic DNA template was incubated at 37 °C with *Eco*RI (New England Biolabs), followed by ligation with adapters that included different barcode-containing adapters for tagging each sample. Ligation was performed using T_4_ DNA ligase (New England Biolabs) followed by heat inactivation at 65 °C. Afterwards, PCR was performed to amplify the bilateral tag sequences. The PCR products were purified using Agencourt AMPure XP (Beckman), pooled, and then run on a 2% agarose gel. Fragments 400–425 bp (with indexes and adapters) in size were isolated using a gel extraction kit (Qiagen). These fragment products were then purified using Agencourt AMPure XP (Beckman), which was diluted for sequencing. Paired-end sequencing was performed on selected tags using the Illumina PE150 high-throughput sequencing platform (Illumina Inc., San Diego, USA) with 2 × 150 bp paired reads. SNP genotyping and evaluation were subsequently carried out.

### Data processing and SNP calling

Sequences for each sample were sorted according to barcodes. To ensure that reads were reliable and without artificial bias in subsequent analyses, we first processed raw data in the fastq format using a series of quality control procedures with in-house C scripts. Briefly, this involved (1) removing reads with ≥10% unidentified nucleotides (N); (2) removing reads with >50% bases possessing a phred quality score <5; and (3) removing reads with >10 nt aligned to adapters, allowing ≤10% mismatches. Subsequently, all reads mapped to the same location were considered a “stack.” SNP sites were identified within each stack using SAMtools^49^ with dp1 and Miss0.6 filters. Polymorphic bases detected among SNPs were supported by at least three reads for each respective SNP.

### Genetic structure analyses

The genetic structure of *Zanthoxylum* cultivars was analyzed using five complimentary methods: neighbor-joining tree analysis, PCA, sNMF^50^, fastSTRUCTURE^51^, and DAPC^52^. These analyses were based on datasets retaining only one random SNP per locus to avoid creating a set of tightly linked sites. Neighbor-joining tree and PCA approaches were applied using the R package poppr^53^. The most likely number of genetic clusters was determined by (1) running sNMF with 1 − *K* assumed ancestral populations using an entropy criterion to fit the statistical model to the data by cross-validation; (2) running fastSTRUCTURE for multiple numbers of *K* using fivefold cross-validation and comparing the log-marginal likelihood lower bound; and (3) running DAPC for multiple numbers of *K* and comparing AIC values. All analyses were repeated after removing loci under strong divergent or stabilizing selection to reflect demographic processes such as gene flow and genetic drift. The repeated analyses resulted in no significant differences. Patterns of variation and population differentiation, including patterns of nucleotide diversity (*π*), heterozygosity, and the fixation index (*F*_*ST*_)^54^, were estimated based on the genotypes of each line at SNP positions using VCFtools^55^.

### Demographic history inference

Single-population demographic histories were investigated using the stairway plot method^56^ with the folded SFS from each clade (estimated as described above). The results were calibrated using a generation time *g* = 4 years based on field observations and a mutation rate *μ* = 2.6 × 10^−9^ substitutions per site per year according to the literature^20^. To study the history of population splitting, we used SFS-based composite likelihood demographic modeling implemented in fastSIMCOAL2^31^. The same method was also used to reconstruct a more recent Z. *armatum* demographic history that could not be estimated by stairway plots due to the very limited amount of variation within this species.

In total, we specified 12 demographic models for *Z*. *armatum* (Supplementary Fig. 5, Models 1–12), which were designed to represent a range of demographic processes, including lineage divergence, population bottlenecks, population expansion or contraction, and gene flow. The same 12 alternative models of historical divergence were also used to simulate the divergence between *Z*. *armatum* and *Z*. *bungeanum* by fitting to the allele-frequency spectrum of these two species (Supplementary Fig. 5, Models 1–12). Only three divergence models among three genetic populations of *Z*. *bungeanum* were considered due to intensive computation for three-branched models, but we incorporated population size changes into each model and allowed migration between all demes for parameter estimation (Supplementary Fig. 5, Models 13–15).

The observed two- and three-dimensional SFS and bootstrap SFS scores for *Z*. *armatum* and *Z*. *bungeanum* were calculated using a Python script (available at https://github.com/isaacovercast/easySFS). We used 100,000 simulations and 50 cycles of the Brent algorithm to maximize the likelihood of the models. A total of 50 independent optimizations were performed to retain the global maximum likelihood model, and the maximized likelihood observed across all iterations was used for model comparison.

### Species distribution modeling

To assess spatial variation in environmental suitability for *Z*. *armatum* and *Z*. *bungeanum*, we constructed correlative maps of potential distributions with the statistical niche modeling algorithm MaxEnt v3.4^57^ using records of species presence and environmental data. The model was projected to past environmental conditions during the mid-Holocene (6000 years before present (y B.P.)), the LGM at 21,000 y B.P. (MIROC4m general circulation model, Pliocene Model Intercomparison Project), and the Last Interglacial at 120,000 y B.P.^58^.

We gathered 522 georeferenced occurrence points (200 for *Z. bungeanum* and 322 for *Z. armatum*) from our fieldwork, herbarium databases (https://www.cvh.ac.cn), and the Global Biodiversity Information Facility (https://www.gbif.org), which were then vetted for spatial and taxonomic accuracy. To reduce the effects of sampling bias on model prediction, we randomly selected one of multiple occurrence records per species within a 10 km radius using SDMtoolbox^59^. The final dataset for SDM building was composed of 133 records of *Z. bungeanum* and 166 records of *Z. armatum*. As background data, we randomly selected 10,000 points over the entire distribution area of each species. We extracted climate information from 19 layers of bioclimatic variables available from the WorldClim website and selected seven uncorrelated variables (Pearson correlation < 0.7) downloaded from Bioclim at a 2.5 arc minute resolution: mean diurnal range, isothermality, minimum temperature of the coldest month, mean temperature of the wettest quarter, precipitation of the driest month, precipitation seasonality, and precipitation of the warmest quarter.

Models were run using the autofeatures function, the default regularization multiplier, and 100-replicate subsampling, with random training test percentages (70% of observations for model training and 30% for model testing). To determine whether the discrimination capacity of models was better than random chance, models were validated by assessing area under the receiver operating curve (AUC), sensitivity, specificity, and accuracy values averaged across replicates. The high mean AUC values (0.926 ± 0.043 for *Z. armatum* and 0.927 ± 0.066 for *Z. bungeanum*, *n* = 100) indicated good predictive power and performance significantly better than that of a random model (AUC = 0.5). The most important variable was minimum temperature of the coldest month (evaluated with 100 iterations).

### Relationships between cultivars

*f*_3_-statistics^60,61^ and *D*-statistics^62^ were used to formally assess relationships between cultivars. These two statistics were computed using the 3PopTest and *qpDstat* programs from the ADMIXTOOLS package, respectively^60^. *Z*-scores were calculated via a block jackknife approach to assess test significance, with *Z*-scores of a magnitude greater than three corresponding approximately to a *p* value < 0.001. Moreover, we used *qpGraph* from the ADMIXTOOLS package to construct the AG framework^61^ in order to explore different models for population separation followed by admixture that might accommodate the patterns of allele frequencies observed in our data. Further, we assessed goodness-of-fit by investigating the correlations in allele-frequency differentiation statistics (*f*-statistics) observed between all pairs, triplets, and quadruplets of populations and tested whether they differed significantly from empirical values.

We started with a skeleton phylogenetic tree consisting of HYDHP and three randomly chosen cultivars, added the remaining cultivars incrementally to all possible edges in the tree using ADMIXTUREGRAPH^60^, and retained only graph solutions yielding a minimum |*Z*| value between empirical and predicted *f*-statistics. To reduce computational load and model-solving difficulties, some genetically similar and geographically close cultivars were merged into one genetic group (e.g., WDDHP composed of WDDHP, CBDHP, YLDHP, and QADHP; WDBYJ composed of WDBYJ, QAYJ, YLYJ, YLBJ, and QABJ; YXHJ composed of YXHJ, ZTHJ, and KMHJ; and HCDHP composed of HCDHP and SZT). We assessed the robustness of the best model and its predictions using pooled *f-*statistics and by fitting the model using cultivars added in different orders.

### Selection scan

Four outlier tests were used to scan for signatures of selection, namely, LEA^63^, OutFLANK^64^, the Fdist method implemented in Arlequin 3.5^65^, and FLK^66^. Tests were performed first on a dataset in which samples were grouped into two populations, representing the two species, to identify outlier loci under divergent selection. Then, tests were carried out on each species separately, and samples were grouped according to the results from genetic analyses, namely, wild and cultivated groups for *Z. armatum* and three major geographic groups for *Z*. *bungeanum*, to look for signatures of spatially diversifying selection. Loci that were identified as outliers by all tests were considered to be under putative selection.

## Supplementary information

suppementary information

## Data Availability

The short sequence reads reported in this paper have been deposited in the GenBank Short Read Archive (BioProject PRJNA382467, Short Read Study SRP199817). The sequences from the outliers’ flanking regions have been deposited in the GenBank database (accession nos. MK989636–MK989659).

## References

[1] Wu GA (2018). Genomics of the origin and evolution of *Citrus*. Nature.

[2] Wang X (2017). Genomic analyses of primitive, wild and cultivated citrus provide insights into asexual reproduction. Nat. Genet..

[3] Xu Q (2013). The draft genome of sweet orange (*Citrus sinensis*). Nat. Genet..

[4] Kubitzki K., Kallunki J., Duretto M., Wilson P. Rutaceae. In *The Families and Genera of Vascular Plants, Flowering Plants Eudicots: Sapindales, Cucurbitales, Myrtaceae* (ed. Kubitzki, K.) 276–356 (Springer Verlag, Berlin, 2011).

[5] Matthias B, Stark TD, Corinna D, Sofie LS, Thomas H (2014). All-trans-configuration in *Zanthoxylum* alkylamides swaps the tingling with a numbing sensation and diminishes salivation. J. Agric. Food Chem..

[6] Zhang M (2017). *Zanthoxylum bungeanum* Maxim. (Rutaceae): a systematic review of its traditional uses, botany, phytochemistry, pharmacology, pharmacokinetics, and toxicology. Int. J. Mol. Sci..

[7] Patiño, L. O. J., Prieto R. J. A. & Cuca S. L. E. *Zanthoxylum* genus as potential source of bioactive compounds. In *Bioactive Compounds in Phytomedicine* (ed. Rasooli, I.) 185–218 (InTech, Rijeka, Croatia, 2012).

[8] Chinese Pharmacopoeia Commission. *Chinese Pharmacopoeia*. 159–160 (Science and Technology Press of Shanghai, Shanghai, 2015).

[9] Chinese Pharmacopoeia Commission. *Chinese Pharmacopoeia*. 149 (Science and Technology Press of Shanghai, Shanghai, 2010).

[10] Chinese Pharmacopoeia Commission. *Chinese Pharmacopoeia*. 275 (Science and Technology Press of Shanghai, Shanghai, 1977).

[11] Tiffney BH (1980). Fruits and seeds of the Brandon Lignite, V. Rutaceae. J. Arnold Arbor..

[12] Zeng J (2000). The cultivated origin and distribution of Chinese pepper (in Chinese). Agr. Hist. China.

[13] Ji Y, Li S, Ho C (2019). Chemical composition, sensory properties and application of Sichuan pepper (*Zanthoxylum* genus). Food Sci. Hum. Wellness.

[14] Li X, Liang G, Guo Q, Shang H (2004). Isoenzyme analysis of main species of cultivated *Zanthoxylum* L. in China (in Chinese). J. Southwest Agric. Univ. (Nat. Sci.).

[15] Medhi K, Sarmah DK, Deka M, Bhau BS (2014). High gene flow and genetic diversity in three economically important *Zanthoxylum* Spp. of upper Brahmaputra valley zone of NE India using molecular markers. Meta Gene.

[16] Zheng H, Li Z, Xue H, Wang D, Sun Y (2009). RAPD analysis of the germplasm resources of *Zanthoxylum bungeanum* (in Chinese). J. Northwest For. Univ..

[17] Feng S (2015). Genetic relationships of Chinese prickly ash as revealed by ISSR markers. Biologia.

[18] Gupta DD, Mandi SS (2013). Species-specific AFLP markers for authentication of *Zanthoxylum acanthopodium* & *Zanthoxylum oxyphyllum*. J. Med. Plants Stud..

[19] Kim YM, Jo A, Jeong JH, Kwon YR, Kim HB (2017). Development and characterization of microsatellite primers for *Zanthoxylum schinifolium* (Rutaceae). Appl. Plant Sci..

[20] Feng S (2017). De novo transcriptome assembly of *Zanthoxylum bungeanum* using Illumina sequencing for evolutionary analysis and simple sequence repeat marker development. Sci. Rep..

[21] Yoshida T, Nagai H, Yahara T, Tachida H (2010). Genetic structure and putative selective sweep in the pioneer tree, *Zanthoxylum ailanthoides*. J. Plant Res..

[22] Nagai H, Yoshida T, Kamiya K, Yahara T, Tachida H (2009). Development and characterization of microsatellite markers in *Zanthoxylum ailanthoides* (Rutaceae). Mol. Ecol. Resour..

[23] Feng S (2016). Phylogenetic relationships among cultivated *Zanthoxylum* species in China based on cpDNA markers. Tree Genet. Genomes.

[24] Appelhans MS, Reichelt N, Groppo M, Paetzold C, Wen J (2018). Phylogeny and biogeography of the pantropical genus *Zanthoxylum* and its closest relatives in the proto-Rutaceae group (Rutaceae). Mol. Phylogenet. Evol..

[25] Tokunaga H (2012). Arabidopsis lonely guy (*LOG*) multiple mutants reveal a central role of the *LOG*‐dependent pathway in cytokinin activation. Plant J..

[26] Excoffier L, Foll M (2011). fastsimcoal: a continuous-time coalescent simulator of genomic diversity under arbitrarily complex evolutionary scenarios. Bioinformatics.

[27] Levy M, Wang Q, Kaspi R, Parrella MP, Abel S (2005). Arabidopsis IQD1, a novel calmodulin-binding nuclear protein, stimulates glucosinolate accumulation and plant defense. Plant J..

[28] Bishopp A (2011). A mutually inhibitory interaction between auxin and cytokinin specifies vascular pattern in roots. Curr. Biol..

[29] Mahonen AP (2006). Cytokinin signaling and its inhibitor *AHP6* regulate cell fate during vascular development. Science.

[30] Immanen J (2016). Cytokinin and auxin display distinct but interconnected distribution and signaling profiles to stimulate cambial activity. Curr. Biol..

[31] Schmulling T (2002). New insights into the functions of cytokinins in plant development. J. Plant Growth Regul..

[32] Nguyen KH (2016). Arabidopsis type B cytokinin response regulators *ARR*1, *ARR*10, and *ARR*12 negatively regulate plant responses to drought. Proc. Natl. Acad. Sci. USA.

[33] Zhu J (2015). Low temperature inhibits root growth by reducing auxin accumulation via *ARR*1/12. Plant Cell Physiol..

[34] Zhao L, Feng S, Tian J, Wei A, Yang T (2018). Internal transcribed spacer 2 (ITS2) barcodes: a useful tool for identifying Chinese *Zanthoxylum*. Appl. Plant Sci..

[35] Hu Y (2018). Genetic structure of cultivated *Zanthoxylum* species investigated with SSR markers. Tree Genet. Genomes.

[36] Zeng L (2019). Whole genomes and transcriptomes reveal adaptation and domestication of pistachio. Genome Biol..

[37] Sun Y, An Z (2005). Late Pliocene-Pleistocene changes in mass accumulation rates of eolian deposits on the central Chinese Loess Plateau. J. Geophys. Res..

[38] Favre A (2015). The role of the uplift of the Qinghai-Tibetan Plateau for the evolution of Tibetan biotas. Biol. Rev..

[39] Qi X (2012). Molecular data and ecological niche modelling reveal a highly dynamic evolutionary history of the East Asian Tertiary relict *Cercidiphyllum* (Cercidiphyllaceae). New Phytol..

[40] Wang L (2018). Genome of wild mandarin and domestication history of mandarin. Mol. Plant.

[41] Salojarvi J (2017). Genome sequencing and population genomic analyses provide insights into the adaptive landscape of silver birch. Nat. Genet..

[42] Ma T (2018). Ancient polymorphisms and divergence hitchhiking contribute to genomic islands of divergence within a poplar species complex. Proc. Natl. Acad. Sci. USA.

[43] Zhou Y, Massonnet M, Sanjak JS, Cantu D, Gaut BS (2017). Evolutionary genomics of grape (*Vitis vinifera* ssp. *vinifera*) domestication. Proc. Natl. Acad. Sci. USA.

[44] Durvasula A (2017). African genomes illuminate the early history and transition to selfing in *Arabidopsis thaliana*. Proc. Natl. Acad. Sci. USA.

[45] Yu Y (2018). Genome re-sequencing reveals the evolutionary history of peach fruit edibility. Nat. Commun..

[46] Beissinger TM (2016). Recent demography drives changes in linked selection across the maize genome. Nat. Plants.

[47] Meyer RS (2016). Domestication history and geographical adaptation inferred from a SNP map of African rice. Nat. Genet..

[48] Pearson PN, Palmer MR (2000). Atmospheric carbon dioxide concentrations over the past 60 million years. Nature.

[49] Li H (2009). The sequence alignment/map format and SAMtools. Bioinformatics.

[50] Frichot E, Mathieu F, Trouillon T, Bouchard G, François O (2014). Fast and efficient estimation of individual ancestry coefficients. Genetics.

[51] Raj A, Stephens M, Pritchard JK (2014). fastSTRUCTURE: variational inference of population structure in large SNP data sets. Genetics.

[52] François B, Sébastien D, Thibaut J (2010). Discriminant analysis of principal components: a new method for the analysis of genetically structured populations. BMC Genet..

[53] Kamvar ZN, Tabima JF, Grunwald NJ (2014). *Poppr*: an R package for genetic analysis of populations with clonal, partially clonal, and/or sexual reproduction. PeerJ.

[54] Weir BS, Cockerham CC (1984). Estimating *F*-statistics for the analysis of population structure. Evolution.

[55] Danecek P (2011). The variant call format and VCFtools. Bioinformatics.

[56] Liu X, Fu Y (2015). Exploring population size changes using SNP frequency spectra. Nat. Genet..

[57] Phillips SJ, Anderson RP, Schapire RE (2006). Maximum entropy modeling of species geographic distributions. Ecol. Model..

[58] Ottobliesner BL, Marshall SJ, Overpeck JT, Miller GH, Hu A (2006). Simulating Arctic climate warmth and icefield retreat in the last interglaciation. Science.

[59] Brown JL, Bennett JR, French CM (2017). SDMtoolbox 2.0: the next generation Python-based GIS toolkit for landscape genetic, biogeographic and species distribution model analyses. PeerJ.

[60] Patterson N (2012). Ancient admixture in human history. Genetics.

[61] Reich D, Thangaraj K, Patterson N, Price AL, Singh L (2009). Reconstructing Indian population history. Nature.

[62] Green RE (2010). A draft sequence of the Neandertal genome. Science.

[63] Frichot E, François O (2015). LEA: an R package for landscape and ecological association studies. Methods Ecol. Evol..

[64] Whitlock MC, Lotterhos KE (2015). Reliable detection of loci responsible for local adaptation: inference of a null model through trimming the distribution of *F*_*ST*_. Am. Nat..

[65] Excoffier L, Lischer HE (2010). Arlequin suite ver 3.5: a new series of programs to perform population genetics analyses under Linux and Windows. Mol. Ecol. Resour..

[66] Bonhomme M (2010). Detecting selection in population trees: the Lewontin and Krakauer test extended. Genetics.

