# Supplementary material for: Genomic analysis reveals the genetic diversity, population structure, evolutionary history and relationships of Chinese pepper
Source: Hortic Res. 2020 Oct 1;7:158. doi: 10.1038/s41438-020-00376-z (PMC7527552; doi:10.1038/s41438-020-00376-z)
Supplement: Supplementary file 1 — suppementary information [file 41438_2020_376_MOESM1_ESM.docx]

Supplement for

Genomic analysis reveals the genetic diversity, population structure, evolutionary history and relationships of Chinese pepper

Shijing Feng^1,3†^, Zhenshan Liu^2†^, Yang Hu^1,3^, Jieyun Tian^1,3^, Tuxi Yang^1,3^ and Anzhi Wei^1,3*^

^1^College of Forestry, Northwest A&F University, Yangling 712100, Shaanxi, China.

^2^College of Life Science, Northwest A&F University, Yangling 712100, Shaanxi, China.

^3^Research Centre for Engineering and Technology of *Zanthoxylum* State Forestry Administration, Yangling 712100, Shaanxi, China

^†^These authors contributed equally to this work.

Author for correspondence:

*Anzhi Wei*

*Tel: +86-029-* *87082211*

*Email: weianzhi@126.com*


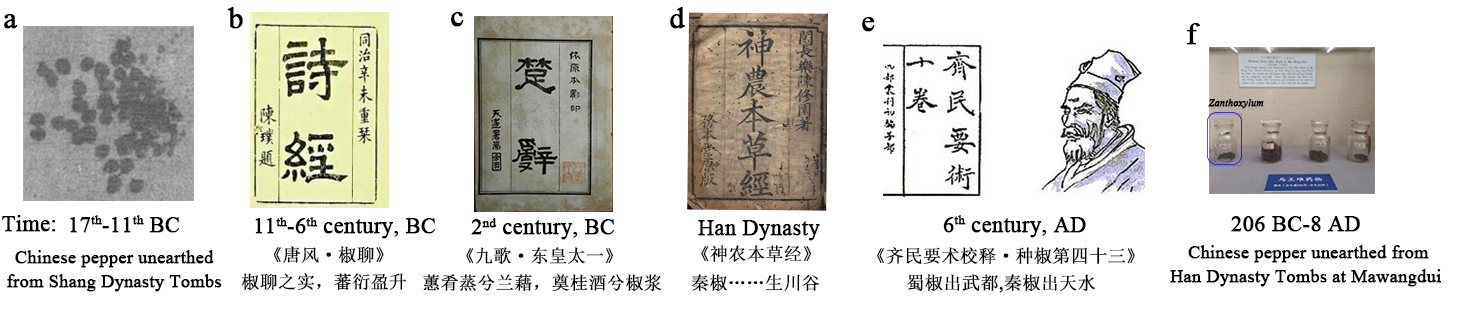


**Supplementary Fig. 1 Historical records of the commercial production of Chinese pepper in China.** **a)**. Chinese pepper unearthed from Shang Dynasty Tombs (*ca.* 17^th^~11^th^ century, BC). **b)**. “JiaoLiao” was a special poem in *Shijing* (Classic of Poetry, *ca.* 11^th^~6^th^ century, BC), which described Chinese pepper as a a precious token and used for worshiping ancestors and praying for more children and a good harvest. **c)**. Chinese pepper was recorded in Qu Yuan’s poem “Nine Songs” as a spice (*ca.* 2nd century, BC). **d)**. *ShenNongBenCaoJing* (Divine Farmer’s Cassic of Materia Medica) recorded that Chinese pepper was used as a traditional Chinese medicine. **e)**. “*Qimin Yaoshu*” (Essential Techniques for the Welfare of the People, an ancient Chinese agricultural texts written by the Northern Wei Dynasty official Jia Sixie) described the origin of cultivation of Chinese pepper occurred in Gansu at least 1,500 years ago in Jin Dynasty. **f)**. Chinese pepper unearthed from Han Dynasty tombs at Mawangdui (ca. 206 BC~8 AD). The pictures of a, b, c, d, e and f were downloaded from public websites (Baidu).


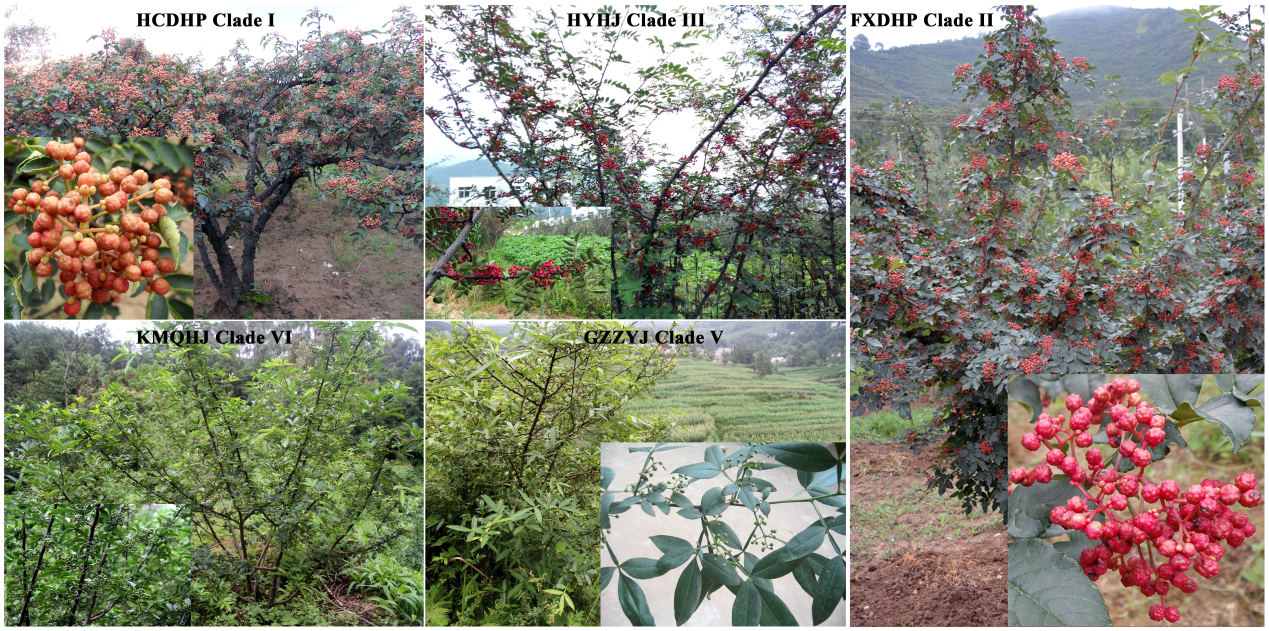


**Supplementary Fig. 2 The representative phenotype characteristics of each clade.** The leaflets of *Z. bungeanum* (Clade I, Clade II and Clade III) are opposite, ovate orbicular or oblongovate and crenulate, and the red pericarp is used as essential cuisine spices. However, the *Z. armatum* (Clade V and Clade VI) are characterized by the presence of green pericarp used in cuisine, odd pinnate compound leaves with winged leaf-stalk. The *Z. bungeanum* clades have great differences in terms of quality and morphological characters. For example, the leaves of HCDHP are narrow and light green, while that of FXDHP are wider, more flat and dark green. The pericarp of HCDHP is thicker, slightly yellow, with less oil glands. The tingling or numbing sensation of HCDHP is relatively strong. However, the pericarp color of FXDHP is bright red and the fruit size is relatively small with well-developed oil glands, leading to a strong fragrance. The yield of HCDHP is relatively high than FXDHP, while the quality of FXDHP is better. The quality of HYHJ is equal to FXDHP while the fruit clusters are strung on the branchlets. The cultivated *Z. armatum* have much increased fruit setting percentage and fruit size compared with wild accessions.


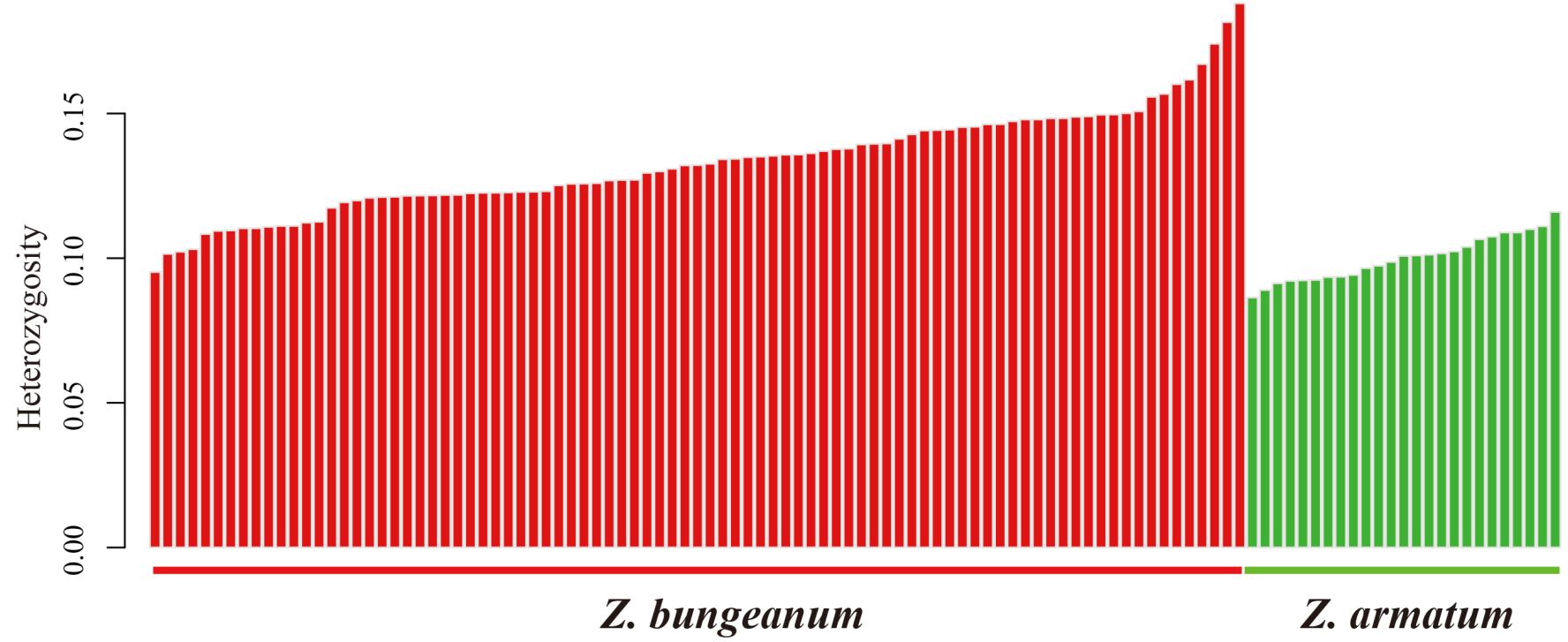


**Supplementary Fig. 3 Heterozygosity of *Z. bungeanum* (red) and *Z. armatum* (green)**

**
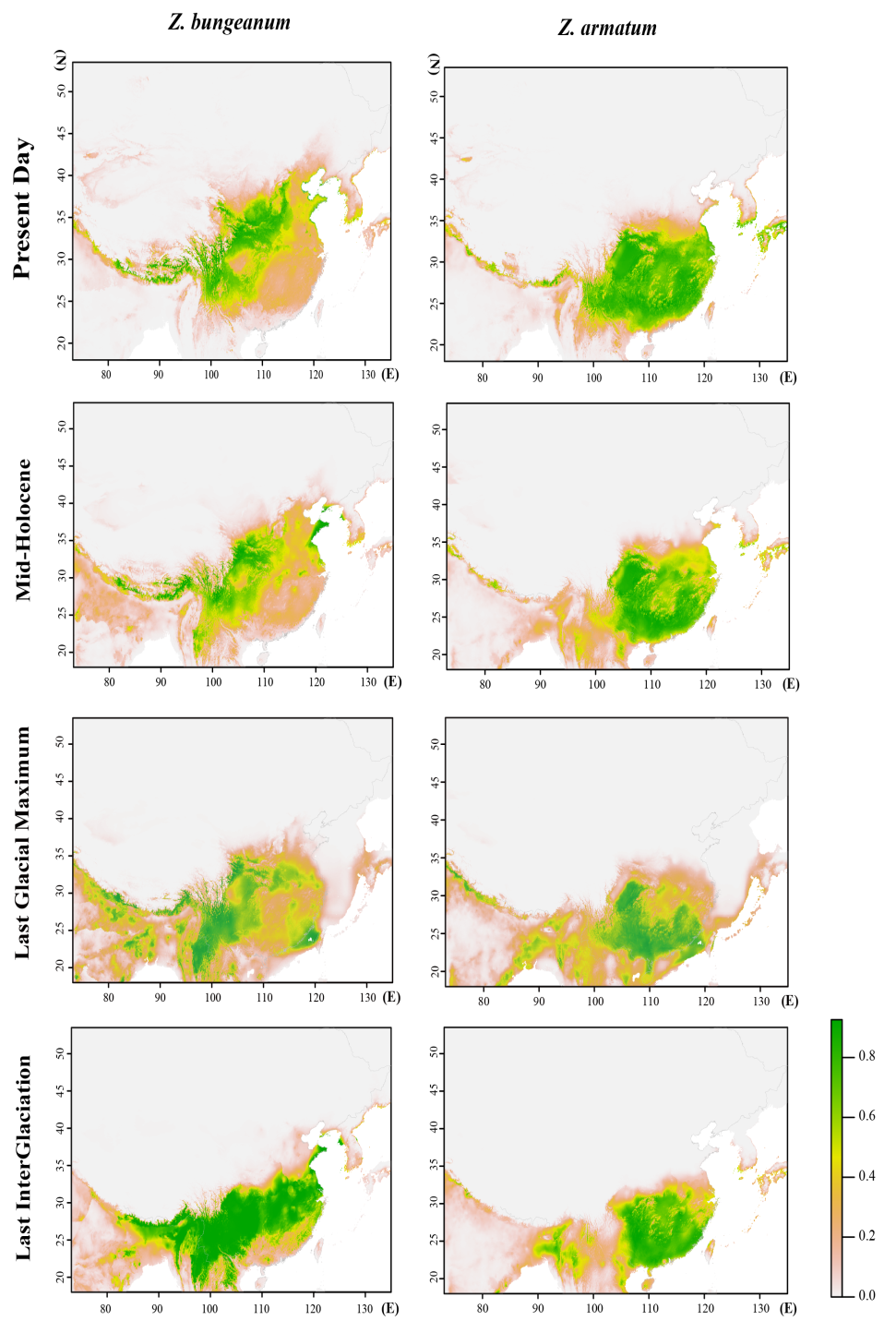
**

**Supplementary Fig. 4 Projections of suitable habitat for *Z. bungeanum* (left) and *Z. armatum* (right).** Shown from upper are estimates of the current ecological niche, as well as projections of this niche onto past conditions of the mid-Holocene, the LGM, and the LIG.


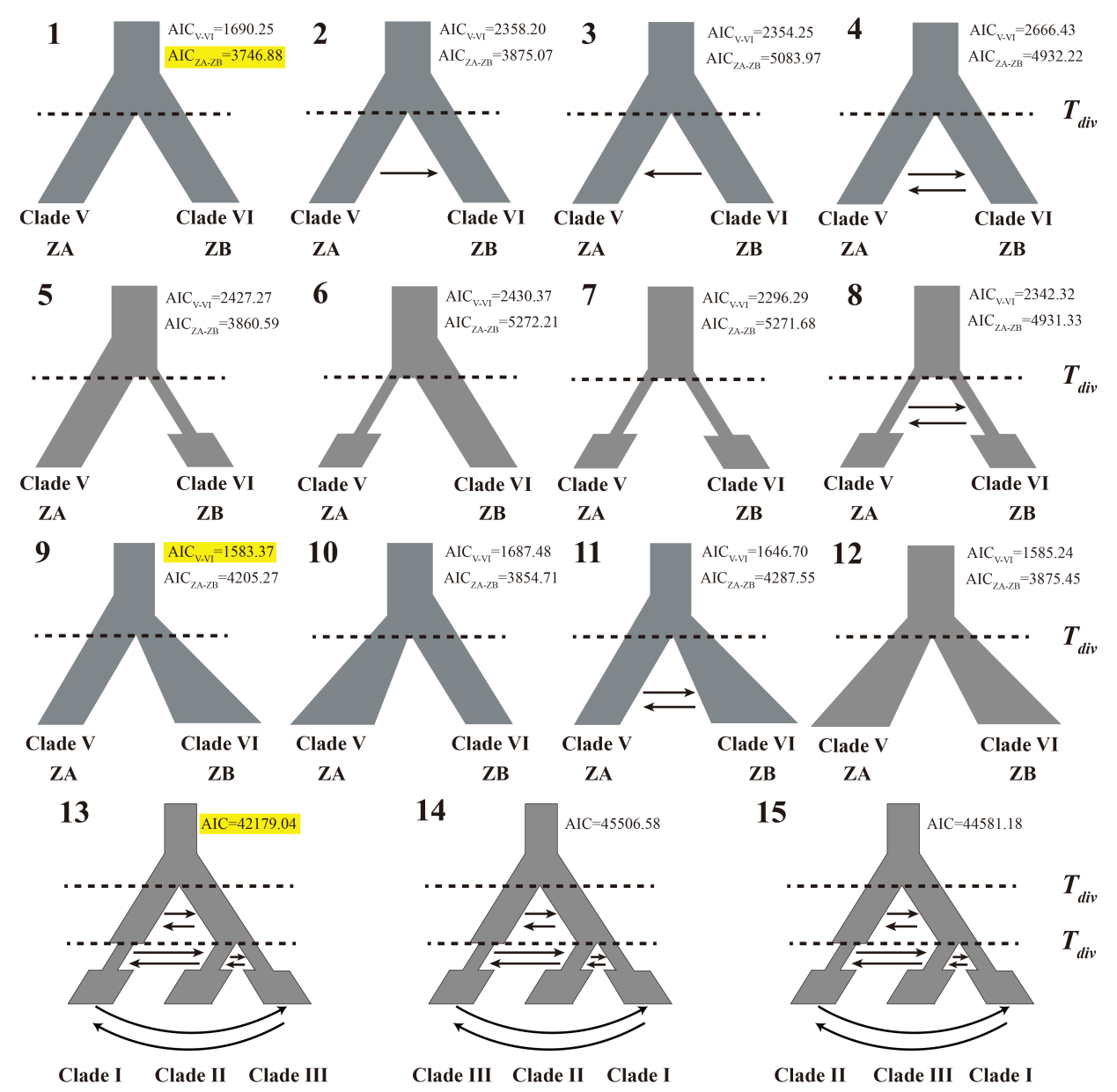


**Supplementary Fig. 5 Fifteen demographic models used in model selection are shown.** The first 12 models (1~12) was used to fit the demographic divergence between *Z. armatum* (ZA) and *Z. bungeanum* (ZB), and between wild and cultivated *Z. armatum* clades. The last three models represented the demographic divergence among *Z. bungeanum* clades. *T_div_* represents the timing of clade divergence. The arrows represent gene flow. ZB: *Z. bungeanum*; ZA: *Z. armatum*. Akaike information criterion (AIC) scorces were shown to rank models, the best ones were colored yellow.


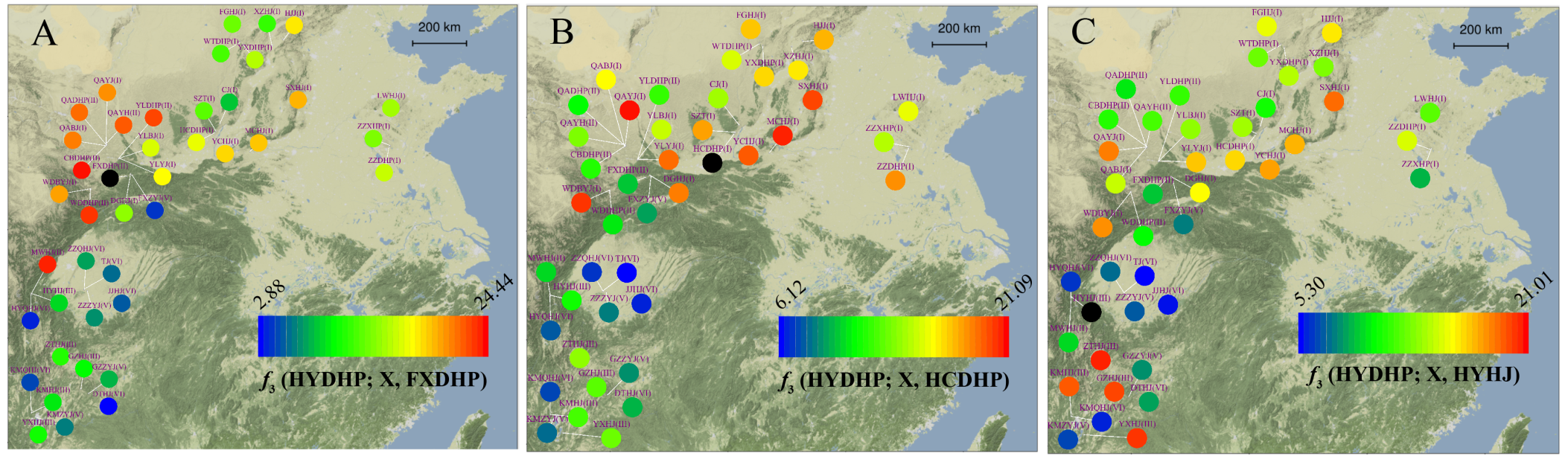


**Supplementary Fig. 6 Genetic affinity of cultivars to FXDHP, HCDHP and HYHJ**, quantified by the out group *f_3_*-statistics of the forms **(A)** *f_3_* (HYDHP; X, FXDHP), **(B)** *f_3_* (HYDHP; X, HCDHP), **(C)** *f_3_* (HYDHP; X, HYHJ), for each cultivar in the genotype panel. Warmer colours represent greater shared drift between a cultivar (X) and FXDHP, HCDHP and HYHJ, respectively.

Supplementary Table 1 Geographic information of *Zanthoxylum* accessions from China surveyed for GBS

| Code | **Cultivar or Wild** | **Abb.** | **Long.** | **Lat.** | **Individuals** | **Location** | **Species** |
| --- | --- | --- | --- | --- | --- | --- | --- |
| 1 | Zaozhuangdahongpao | ZZDHP | 117.68 | 34.94 | 2 | Zaozhuang, Shandong | *Z. bungeanum* |
| 2 | Zaozhuangxiaohongpao | ZZXHP | 117.73 | 34.98 | 3 |  |  |
| 3 | Laiwuhuajiao | LWHJ | 117.67 | 36.21 | 3 | Laiwu, Shandong |  |
| 4 | Xinzhouhuajiao | XZHJ | 112.7 | 38.4 | 2 | Xinzhou, Shanxi |  |
| 5 | Huangjinjiao | HJJ | 113.17 | 38.61 | 3 | Wutai, Shanxi |  |
| 6 | Wutaidahongpao | WTDHP | 111.34 | 38.54 | 2 |  |  |
| 7 | Yuxiandahongpao | YXDHP | 111.48 | 38.45 | 4 | Yuxian, Shanxi |  |
| 8 | Fuguhuajiao | FGHJ | 111.07 | 39.03 | 3 | Fugu, Shaanxi |  |
| 9 | Shexianhuajiao | SXHJ | 113.61 | 36.60 | 1 | Shexian, Hebei |  |
| 10 | Yunchenghuajiao | YCHJ | 110.7 | 34.70 | 1 | Ruicheng, Shanxi |  |
| 11 | Choujiao | CJ | 110.57 | 35.74 | 1 | Hancheng, Shaanxi |  |
| 12 | Hanchengdahongpao | HCDHP | 110.27 | 35.44 | 6 |  |  |
| 13 | Shizitou | SZT | 110.31 | 35.49 | 2 |  |  |
| 14 | Mianchidahongpao | MCHJ | 111.76 | 34.77 | 1 | Mianchi, Henan |  |
| 15 | German huajiao | DGHJ | 106.67 | 33.99 | 3 | Fengxian, Shaanxi |  |
| 16 | Fengxiandahongpao | FXDHP | 106.51 | 33.91 | 3 |  |  |
| 17 | Wudubayuejiao | WDBYJ | 104.99 | 33.46 | 4 | Wudu, Gansu |  |
| 18 | Wududahongpao | WDDHP | 104.97 | 33.46 | 5 |  |  |
| 19 | Yuanlongbaijiao | YLBJ | 106.16 | 34.53 | 1 | Yuanlong, Gansu |  |
| 20 | Yuanlongyoujiao | YLYJ | 106.19 | 34.54 | 1 |  |  |
| 21 | Yuanlongdahongpao | YLDHP | 106.15 | 34.52 | 4 |  |  |
| 22 | Qin'anbaijiao | QABJ | 105.61 | 34.98 | 2 | Qin'an, Gansu |  |
| 23 | Qin'anyoujiao | QAYJ | 105.63 | 35.02 | 2 |  |  |
| 24 | Qin'andahongpao | QADHP | 105.65 | 35.00 | 2 |  |  |
| 25 | Changbingdahongpao | CBDHP | 105.67 | 35.03 | 1 |  |  |
| 26 | Qin'anyihao | QAYH | 105.63 | 35.05 | 5 |  |  |
| 27 | Maowenhuajiao | MWHJ | 102.45 | 29.65 | 2 | Hanyuan, Sichuan |  |
| 28 | Hanyuanhuajiao | HYHJ | 102.46 | 29.66 | 5 |  |  |
| 29 | Hanyuandahongpao | HYDHP | 102.56 | 29.76 | 3 |  |  |
| 30 | Guizhouhuajiao | GZHJ | 104.56 | 26.07 | 4 | Sajizhen, Guizhou |  |
| 31 | Kunminghuajiao | KMHJ | 102.45 | 25.15 | 3 | Kunming, Yunnan |  |
| 32 | Yuxihuajiao | YXHJ | 102.98 | 24.44 | 2 | Qinglong, Yunnan |  |
| 33 | Zhaotonghuajiao | ZTHJ | 103.7 | 27.3 | 1 | Zhaotong, Yunnan |  |
| 34 | Zhuyehuajiao_Wild | FXZYJ | 106.73 | 33.95 | 4 | Fengxian, Shaanxi | *Z. armatum* |
| 35 | Zhuyehuajiao_Wild | GZZYJ | 104.89 | 25.9 | 4 | Baojixiang, Guizhou |  |
| 36 | Hanyuanqinghuajiao | HYQHJ | 102.44 | 29.63 | 3 | Hanyuan, Sichuan |  |
| 37 | Jiangjinhuajiao | JJHJ | 106.30 | 29.3 | 1 | Jiangjin, Chongqing |  |
| 38 | Zhuyehuajiao_Wild | ZZZYJ | 104.83 | 29.76 | 2 | Zizhong, Sichuan |  |
| 39 | Tengjiao | TJ | 104.69 | 29.82 | 1 |  |  |
| 40 | Zizhongqinghuajiao | ZZQHJ | 106.70 | 29.80 | 2 |  |  |
| 41 | Dingtanhuajiao | DTHJ | 105.66 | 25.68 | 4 | Guanling, Guizhou |  |
| 42 | Kunmingqinghuajiao | KMQHJ | 102.45 | 25.15 | 2 | Kunming, Yunnan |  |
| 43 | Zhuyehuajiao_Wild | KMZYJ | 102.60 | 25.00 | 2 |  |  |
| Total |  |  |  |  | **112** |  |  |

List of Abbreviations

C.E.: Common Era

GBS: Genotyping-by-sequencing

SNP: Single Nucleotide Polymorphism

NGS: Next Generation Sequencing

PCA: Principal Component Analysis

sNMF: Sparse Nonnegative Matrix Factorization

DAPC: Discriminant Analysis of Principal Components

SFS: Skews in Site Frequency Spectrum

LGM: Last Glacial Maximum

LIG: Last Interglacial

SDM: Species Distribution Modeling

AIC: Akaike Information Criterion

CI: Confidence Interval

AG: Admixture Graph

GBIF: Global Biodiversity Information Facility

AUC: the area under the receiver operating curve

kya: thousand years ago

mya: million years ago

*g*: generation time

*μ*: mutation rate per site per year

*π*: nucleotide diversity

*F_ST_*: genetic differentiation statistics

*N_e_*: effective population size
